# Sleep Deprivation in Rats Causes Dissociation of the Synaptic NMDA Receptor/D1 Dopamine Receptor Heterocomplex

**DOI:** 10.3390/neurosci6030061

**Published:** 2025-07-05

**Authors:** Natalia Kiknadze, Nana Narmania, Maia Sepashvili, Tamar Barbakadze, Elene Zhuravliova, Tamar Shetekauri, Nino Tkemaladze, Nikoloz Oniani, David Mikeladze

**Affiliations:** 1 Institute of Chemical Biology, Ilia State University, 3/5 Cholokashvili av., Tbilisi 0160, Georgia; natalia.kiknadze.1@iliauni.edu.ge (N.K.); nana.narmania@iliauni.edu.ge (N.N.); maia.sepashvili@iliauni.edu.ge (M.S.); tamar@barbakadze.com (T.B.); elene_zhuravliova@iliauni.edu.ge (E.Z.); tamar.shetekauri.1@iliauni.edu.ge (T.S.); nino.tkemaladze.5@iliauni.edu.ge (N.T.); nikoloz.oniani@iliauni.edu.ge (N.O.); 2 I.Beritashvili Center of Experimental Biomedicine, 14 Gotua St., Tbilisi 0162, Georgia

**Keywords:** sleep deprivation, synaptic plasticity, glutamate receptors, Homer

## Abstract

Glutamate and dopamine receptors play a crucial role in regulating synaptic plasticity throughout the sleep–wake cycle. These receptors form various heterocomplexes in synaptic areas; however, the role of this protein interactome in sleep–wake cycles remains unclear. Co-immunoprecipitation experiments were conducted to observe the complexation of the NMDA glutamate receptor (NMDAR) subunits GluN2A and GluN2B, metabotropic glutamate receptors mGluR1/5, and dopamine receptors (D1R and D2R) with the scaffold protein Homer in the synaptic membranes of the hippocampus after six hours of sleep deprivation (SD) in rats. Our findings indicate that the level of Homer in the GluN2A/mGluR1/D1R interactome decreased during SD, while the content of Homer remained unchanged in the GluN2B/mGluR1/D2R heterocomplex. Moreover, Homer immunoprecipitated a reduced amount of inositol trisphosphate receptor (IP3R) in the microsomal and synaptic fractions, confirming the dissociation of the ternary supercomplex Homer/mGluR1/IP3R during SD. Additionally, our findings indicate that SD increases the synaptic content of the AMPA receptor (AMPAR) subunit GluA1. Unlike AMPAR, NMDAR subunits in synaptic membranes do not undergo significant changes. Furthermore, the G-to-F actin ratio decreases during SD. Changes in the assembly of actin filaments occur due to the dephosphorylation of cofilin. These results suggest that SD causes the dissociation of the GluN2A/mGluR1/D1R/Homer/IP3R heterocomplex in synaptic and endoplasmic membranes.

## 1. Introduction

Sleep is closely linked to changes in synaptic plasticity, a process that plays a crucial role in memory formation and learning. During sleep, synaptic connections either strengthen or weaken by activating or inhibiting various neurotransmitter receptors, significantly contributing to memory consolidation [1]. Glutamatergic signaling is crucial for regulating sleep and wakefulness, with the activity of both metabotropic and ionotropic glutamate receptors being pivotal in managing synaptic plasticity and memory consolidation throughout the sleep–wake cycle. AMPA and NMDA receptors play a crucial role in modulating sleep–wake cycles and initiating sleep stages, although their exact roles in synaptic plasticity remain under investigation [2]. In addition to ionotropic receptors (AMPAR and NMDAR), group 1 metabotropic glutamate receptors (mGluR1/5) are particularly important in brain regions that regulate sleep–wake states. Recent research indicates that mGluR1/5 may play a role in sleep homeostasis, maintaining an optimal balance between wakefulness and sleep [3,4]. After sleep deprivation, there is an increase in glutamate levels and an elevation in mGluR1/5 density, suggesting a potential role in facilitating the brain’s transition to sleep and restoring balance [5,6]. Additionally, the activity of mGluR1/5 may help the brain compensate and maintain certain functions during periods of deprivation.

Dopamine (DA), a neurotransmitter associated with movement, motivation, and reward processing, also plays a critical role in learning and memory [7,8]. The dynamics of dopaminergic neuronal activity are essential for regulating sleep stages [9,10]. Sleep deprivation can lead to behavioral changes, such as hyperactivity, increased social and sexual behaviors, and decreased depressive-like symptoms, all influenced by dopamine [11]. Interestingly, SD is linked to dopamine-dependent protein synthesis and an increase in dopaminergic activity, and it promotes dendritic spinogenesis in the prefrontal cortex [12]. During spatial learning, blocking D1/D5 dopaminergic receptors disrupts memory retention [13] and inhibits novelty-facilitated synaptic plasticity [14]. Notably, DA receptors can facilitate allosteric interactions with other receptor proteins, including glutamate receptors, creating multi-receptor complexes that help modulate signal decoding at the membrane level, characterized by specific pharmacological profiles [15].

Most synaptic neurotransmitter receptors are dynamically concentrated at the postsynaptic density (PSD), where they are trafficked and stabilized at the postsynaptic membrane by scaffolding proteins containing PDZ domains, such as GRIP, PICK, and the PSD-95 family, to control synaptic strength [16]. The Homer scaffolding protein family (Homer1–3) is a critical component of the postsynaptic density (PSD) and forms connections between receptors, ion channels, and other scaffolding proteins [17]. These proteins facilitate the assembly of multiprotein complexes in intracellular microdomains and create postsynaptic protein interactomes. Homer is an adaptor protein that primarily interacts with mGluR1/5. Homer proteins are classified into two main types: the long form (Homer1b and c), which is consistently present in the brain, and the short form (Homer1a), a spliced variant of the Homer protein, which is rapidly produced in response to neuronal activity. Research in mice suggests that Homer is essential for prolonged wakefulness [18]. Mice lacking Homer find it challenging to sustain wakefulness and experience fragmented sleep patterns. The expression of Homer1a initiates crucial processes for PSD remodeling, modulates functions related to glutamate receptors, and regulates calcium signaling. It is proposed that the scaffold remodeling induced by Homer1a may switch the mGluR1/5-NMDA heterocomplex and calcium signaling activity in response to synaptic stimulation [18]. This mechanism could be significant for initiating plasticity processes such as long-term potentiation (LTP) and long-term depression (LTD). These findings indicate that short Homer proteins can temporarily destabilize the PSD, thereby allowing scaling of the most current or active synapses. They may also selectively enhance the plasticity of excitatory synapses, thereby supporting critical processes like learning and memory [19]. Furthermore, Homer may facilitate the homeostatic scaling down of excitatory synapses during sleep by regulating calcium levels in neurons [20]. However, the precise interactions of Homer proteins with other proteins during sleep have not yet been fully clarified. Investigating protein interactions during particular stages of sleep could provide further insights into the distinct functions of Homer proteins across different sleep phases. A comprehensive understanding of these interactions is crucial for unraveling the complex mechanisms underlying sleep regulation and may pave the way for the development of future therapies for sleep disorders.

Proteomic approaches can significantly enhance our understanding of the molecular processes underlying sleep and brain cognitive function [21]. Quantitative studies on synaptic structures, including synaptic membranes and postsynaptic densities, can yield valuable insights. These studies track changes in protein abundance and post-translational modifications, enhancing our understanding of circadian clock-driven mechanisms and physiological sleep [22]. Sleep deprivation and related processes have a significant impact on synaptic proteins in both presynaptic and postsynaptic regions. In particular, the abundance of 78 synaptic proteins has been notably altered due to sleep deprivation, with the most considerable changes observed in neurotransmitter receptors, scaffold proteins, and synaptic organizer proteins [23]. These processes are regulated through protein complexes, primarily influenced by protein–protein interactions (PPI) [24,25,26]. Despite extensive research into the pathophysiological mechanisms of sleep deprivation across various models, the molecular changes in the brain associated with forming protein interactomes remain unclear.

In this study, we observed differences in the complexation of Homer scaffold proteins with glutamate and dopamine receptors in the hippocampus following six hours of sleep deprivation in rats. Our findings suggest that heterocomplexes involving glutamate and dopamine receptors change during this period. We propose that Homer proteins are essential for reorganizing the multi-receptor system at the synaptic membranes during sleep-dependent memory consolidation.

## 2. Materials and Methods

### 2.1. Animals

Wistar rats were sourced from the vivarium colony at Ilia State University, Tbilisi, Georgia. Eight male Wistar rats weighing 180–300 g were selected for further analysis. The animals were housed in groups under optimal temperature conditions (22 ± 2 °C) with a 12 h light/dark cycle and ad libitum access to water and food. Before the experiment began, the selected rats were placed in separate cages. During the experiment, food and water provision were not reduced.

### 2.2. Sleep Deprivation

During the experiments, the rats underwent 6 h of total sleep deprivation. The “gentle handling” method was chosen to achieve total sleep deprivation. With the gentle handling method, the total sleep deprivation procedure is undergone following the researcher’s direct intervention. The experimenter keeps the study object awake and prevents it from falling asleep, even if it enters a drowsy state or assumes a resting position. This method of sleep deprivation is widespread, as the study object is already adapted to the cage, and the minimization of stress and concomitant events, such as 18 forced locomotor activities, is considered [27]. Male Wistar rats, weighing approximately 200–250 g, were used in the study. The rats were maintained at a stable temperature of 22 ± 3 °C and exposed to a 12 h light/dark cycle. The animals for the experiments were selected at random. Deprivation began at 9:00 AM and continued for six hours, concluding at 3:00 PM. The animals remained awake for almost the entire period. The brains of both sleep-deprived and undisturbed control animals were removed immediately after the deprivation period as per the AVMA Guidelines for the Euthanasia of Animals, 2020 Edition. The brain structures were extracted from the rat brains and stored on ice.

### 2.3. Subcellular Fractioning

The experiments involved the brain structures of four control male rats and four male rats undergoing sleep deprivation. Subcellular fractions were prepared following the methodology outlined by Won et al. [28]. Immediately after isolation, the brain structures were homogenized using a Dounce homogenizer in an ice-cold buffer containing 10 mM Tris-HCl (pH 7.5), 5 mM EDTA, 1 mM DTT, 1 mM PMSF (TEVP buffer), and a protease inhibitor cocktail, along with 320 mM sucrose. All subsequent procedures were carried out at a temperature of 4 °C. The homogenate was first centrifuged at 1000× *g* for 10 min, and the resulting supernatant was subjected to a second centrifugation at 12,000× *g* for 20 min. The supernatant served as the “cytoplasmic fraction”, while the pellet obtained was resuspended in double the volume of TEVP buffer containing 35.6 mM sucrose. The mixture was then incubated for 30 min and subsequently centrifuged at 25,000× *g* for 20 min. To isolate the extra-synaptic (non-PSD enriched) fraction, the pellet was resuspended in three times its volume of ice-cold TEVP buffer containing 1% Triton X-100. This mixture was allowed to solubilize at 4 °C for 15 min, followed by centrifugation at 20,000× *g* for 30 min. The supernatant obtained from this step was designated as the “extra-synaptic protein fraction”. To extract the synaptic (PSD-enriched) protein fraction, the pellet was resuspended in three times its volume of TEVP buffer containing 1% SDS and incubated for 2 h at 4 °C with gentle vortexing. The final step involved centrifugation at 100,000× *g* for 30 min, after which the supernatant was collected and stored as the “synaptic protein fraction”. All solubilized proteins were preserved at −80 °C until further analysis.

The microsomal fraction (containing endoplasmic reticulum membranes) was obtained from the “cytoplasmic” fraction by centrifugation at 100,000× *g* at 4 °C for 1 h. The resulting pellet was supplemented with solubilization buffer (20 mM HEPES, 150 mM NaCl, 10 mM EDTA, 2 mM EGTA, 1% CHAPS, 0.1% SDS, 1% Triton-100, 0.5% IGEPAL, 0.1% BSA, pH 7.5) to extract the membrane proteins. Resuspension and incubation at 4 °C for 30 min were followed by centrifugation at 20,000× g at 4 °C for 12 min to achieve final fragmentation of the fraction. The obtained supernatant was aliquoted and stored at −80 °C for further analysis.

The protein amounts in the obtained fractions were determined using a BCA protein assay kit (sc-2322, Santa Cruz Biotechnology, Dallas, TX, USA) according to the manufacturer’s protocol. Protein optical absorption was measured using a spectrophotometer at 562 nm.

### 2.4. Immunoprecipitation

Protein A/G Agarose (sc-2003, Santa Cruz, Dallas, TX, USA) was dissolved in lysis buffer (20 mM Hepes, 100 mM NaCl, 1 mM DTT, 1 mM EDTA, 1 mM EGTA, 0.5% Triton X-100 at pH 7.2) and incubated separately with Homer and GluN2a or GluN2b primary antibodies for 2 h at 4 °C with gentle shaking. All samples were adjusted to an equal amount of protein and incubated with the primary antibodies and Protein A/G Agarose mixture for 24 h at 4 °C with gentle shaking. Following incubation, the samples were centrifuged for 10 min at 1000× *g*. Then 100 mM glycine buffer was added to the pellet of each sample. The solute was gently shaken for 3 min at 4 °C and then centrifuged for 5 min at 10,000× *g*. The supernatant from each sample was transferred to microtubes containing pre-added Tris-HCl buffer (pH 9.5). The prepared samples were then used for Western blotting.

### 2.5. Western Blotting

The “Western blot protocol” (Abcam) [29] was used for the Western blot analysis. Aliquots of the synaptic/extra-synaptic plasma membrane protein fractions and microsomal and cytosolic protein fractions (containing approximately 40–60 μg of total protein) were loaded onto SDS gels (4–12%) and electrophoresed after being dissolved in equal amounts. Electroblotting was then employed to transfer the isolated proteins onto 0.45 μm nitrocellulose membranes. Ponceau S solution was used to stain the membranes, confirming proper sample loading and effective protein transfer. After blocking the membranes with 5% BSA in Tris-buffered saline with 0.1% Tween-20 (TBST), the membranes were incubated with the following antibodies against the relevant antigens for one hour.

The following antibodies were used: AMPA receptor subunit GluA1 [sc-13152], NMDA NR1 [sc-518053], p-Coffilin-1 [sc-271921], Coffilin-1 [sc-53934]), beta-actin [sc-32273]; Homer-1 [ab-184955], RhoA [A308049], GluN2A [sc-1468], GluN2B [sc-1469], D1 dopamine receptor [sc-33666], D2 dopamine receptor [sc-5303], IP3R [sc-377518], mGluR1 [sc-99040], mGluR5 [sc-47147]; Santa Cruz Biotechnology Inc. (Dallas, TX, USA) supplied all primary antibodies. Following incubation, the membranes were rinsed in TBST (20 mM Tris, 150 mM NaCl, 0.1% Tween 20, pH 7.5) and probed for one hour at 20 °C using species-appropriate secondary antibodies coupled to peroxidase. These antibodies were detected using enhanced chemiluminescence autoradiography (ECL kit, sc-2048; Santa Cruz, Dallas, TX, USA) after further washing in TBST. β-Actin served as a loading control. After chemiluminescence visualization, the nitrocellulose membrane was treated with stripping buffer (1 L: 15 g glycine, 1 g SDS, 10 mL Tween 20, pH = 2.2) and re-probed for β-actin detection. The membranes were incubated with fresh stripping buffer for 10 min at room temperature, and this process was repeated. The membrane was washed twice in PBS for 10 min. All procedures were conducted at room temperature. The stripped and washed membrane was blocked in a 5% bovine serum albumin TBST solution for western blotting. The autoradiograph films (Amersham) were used to expose the blots. After digitizing the obtained films using photographic equipment, Image Lite Studio software version 5.2.5 (Li-Cor) was employed to quantify the intensities of the films.

### 2.6. F-Actin and G-Actin Fractionation

Fractionation was performed according to the method described by [30], with some modifications. The samples were homogenized in the F-actin stabilization buffer composed of 0.1 M 1,4-piperazinediethanesulfonic acid (PIPES, sc216099, ChemCruz, Dallas, TX, USA), 30% glycerol (G7893, Sigma-Aldrich, USA), 5% dimethyl sulfoxide (DMSO, D8418, Sigma-Aldrich, USA), 1 mM MgSO4 (39773.01, Serva, Heidelberg, Germany), 1 mM EGTA (324628, Millipore, USA), 1% Triton X-100 (X100, Sigma-Aldrich, St. Louis, MO, USA), 1 mM adenosine triphosphate (ATP, A1852, Sigma-Aldrich, St. Louis, MO, USA), and protease inhibitor (P8340, Sigma-Aldrich, St. Louis, MO, USA). Samples were incubated at 37  °C for 10 min and subsequently centrifuged at 100,000× *g* at 37 °C for one hour in an ultracentrifuge (CS150NX, Himac, Hitachinaka, Ibaraki, Japan) to separate G-actin (in the supernatant) from F-actin (in the pellet) fractions. Actin proteins were detected in both fractions by sodium dodecyl sulfate-polyacrylamide gel electrophoresis (SDS-PAGE) and Western blotting using an anti-β-actin antibody (sc-47778, Santa Cruz, Dallas, TX, USA). The band was analyzed using ImageJ (1.53 p, National Institute of Health, Bethesda, MD, USA) software.

### 2.7. Statistical Analysis

Statistical analysis was performed using one-way ANOVA with post hoc Tukey tests to compare multiple treatments. A value of *p* < 0.05 was considered statistically significant.

## 3. Results

**Figure 1:**
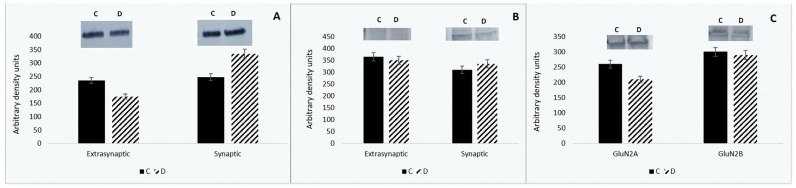
Distribution of the GluA1 subunit of AMPAR (**A**) and the NR1 (**B**) in synaptic and extra-synaptic fractions, and the changes in GluN2A and GluN2B subunits of NMDAR (**C**) in the synaptic fraction in the hippocampus of control (n = 4) and sleep-deprived rats (n = 4). D—Western blot; E—densitometric analyses. Results from triplicate experiments are presented as mean ± SEM. Error bars represent one standard error of the mean; D (sleep deprivation) vs. C (control). Data are normalized and expressed as mean optical density ± standard error of the mean (SEM) (*p* < 0.05).

Ionotropic glutamate receptors in the postsynaptic membrane are essential for neural circuits and information processing in the central nervous system [31]. These receptors exhibit dynamic lateral mobility between the synaptic membrane and the postsynaptic density [32,33,34]. Therefore, we first examined the distribution of AMPA and NMDA receptors in synaptic and extra-synaptic fractions following sleep deprivation (SD). Our findings indicate that six hours of SD increases the synaptic content of the AMPA receptor subunit GluA1, while its amount decreases in extra-synaptic fractions. Unlike AMPA receptors, the levels of NMDAR subunits, including GluN1, GluN2A, and GluN2B, remain stable in both synaptic and extra-synaptic membranes after SD (Figure 1A–C).

**Figure 2:**
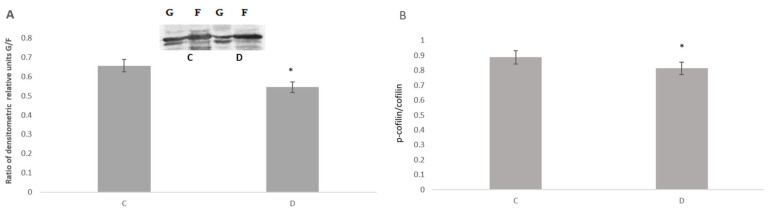
Western blot analysis of the G-to-F actin and phosphorylated cofilin to cofilin1 ratio in the hippocampal cells of control (n = 4) and sleep-deprived rats (n = 4) (**A**). Western blots of the G and F actin bands and densitometric analysis of the G/F ratio (**B**). Diagram showing the analysis of the phosphorylated cofilin to cofilin1 ratio. Results of triplicate experiments are presented as mean ± SEM. Error bars represent one standard error of the mean, and asterisks indicate statistically significant differences: * *p* < 0.05; D (sleep deprivation) vs. C (control).

**Figure 3:**
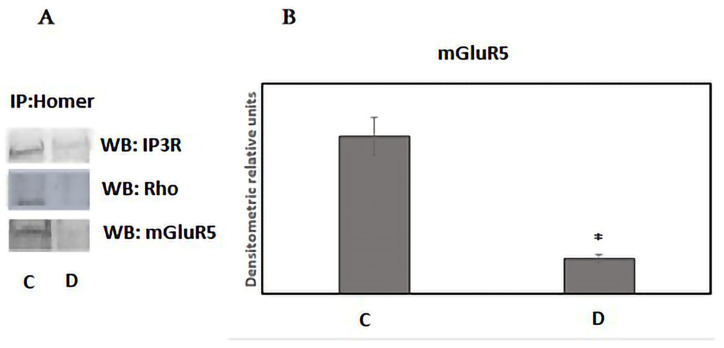
Western blot analysis of proteins bound to anti-Homer antibodies in the microsomal fraction from hippocampal cells of control (n = 4) and sleep-deprived rats (n = 4). (**A**) Western blotting, (**B**) diagram showing the densitometric analysis of the mGluR1/5 in the microsomal fraction from hippocampal cells of control and sleep-deprived rats. Error bars represent one standard error of the mean, and ± standard error of the mean (SEM) (‡ *p* < 0.05); D (sleep deprivation) vs. C (control).

Both forms of long-term synaptic plasticity, LTP and LTD, involve remodeling the actin cytoskeleton within dendritic spines. Multiple studies have indicated spine enlargement during LTP and shrinkage or loss of spines during LTD [35,36]. The initial signaling events activate pathways that regulate the trafficking of AMPA receptors, modulating the dynamics of the actin cytoskeleton in spines, which leads to enhanced postsynaptic membrane expression of AMPA-type glutamate receptors and transient enlargement of dendritic spines [37,38,39]. Based on these observations, the actin cytoskeleton dynamics during sleep deprivation were studied. To assess the extent of actin remodeling, we measured the globular (G) and filamentous (F) actin levels in hippocampal cell fractions. We found that the G-to-F actin ratio decreases with sleep deprivation, indicating the polymerization of the actin cytoskeleton (Figure 2A,B). Changes in the organization of actin filaments occur due to the activation of the regulatory protein cofilin, which undergoes dephosphorylation during sleep deprivation [40]. We also evaluated the levels of actin polymerization-regulating proteins Rac and Rho. These small GTP-binding proteins control the assembly and disassembly of the actin cytoskeleton in response to extracellular stimuli. Our results indicate that only Rho, which was preferentially associated with the Homer-formed interactome, decreased significantly during sleep deprivation (Figure 3A). Therefore, the Rho-regulated and cofilin activation signaling pathway increases hippocampal spine density during sleep deprivation. These findings suggest that sleep deprivation promotes assembly in the actin cytoskeleton, enhancing the dynamics and mobility of postsynaptic elements.

Homer1, an essential component of the excitatory postsynaptic scaffolding complex, has been shown to modulate excitatory synaptic plasticity by regulating type 1 metabotropic glutamate receptors (mGluR1/5) [20]. Different isoforms of Homer, including long-form and short-form variants, are implicated in sleep functions. During wakefulness, long-form Homer protein tethers synaptic mGluR1/5 to endoplasmic inositol trisphosphate receptors (IP3R) and the postsynaptic density, significantly impacting synaptic plasticity and influencing cognitive processes. This heterocomplex formation ensures a quantal release of calcium from the ER in response to synaptic glutamate activity.

Immunoprecipitation experiments were conducted to assess changes in the components of the ternary complex of mGluR5/IP3R/Homer during sleep deprivation. We evaluated the association between mGluR5 and IP3R with Homer in the endoplasmic microsomal membranes. Our findings revealed that Homer complexes significantly less with mGluR5 in the microsomal fraction during deprivation compared to the control state (Figure 3A,B). Additionally, Homer immunoprecipitated a lower amount of IP3R in the sleep-deprived animals, confirming the dissociation of the ternary supercomplex during sleep deprivation. These results support the hypothesis that sleep deprivation induces the dissociation of the heterocomplex involving the IP3R, Homer1, and mGluR5 receptors, which may lead to a decrease in the glutamate-dependent efflux of calcium from the endoplasmic reticulum [20]. Consequently, sleep deprivation leads to the dissociation of the supercomplex comprising IP3R, Homer1, and mGluR5, situated between synaptic and endoplasmic membranes.

Several types of glutamate and dopamine receptors form heterocomplexes in synaptic and extra-synaptic areas, influencing the signaling from individual protomers [15,41]. Research indicates that prolonged dopamine stimulation reduces the D1 and D2 dopamine receptors within the heterocomplex [42]. This reduction is likely due to the transient interaction between beta-arrestin-2 and the adenosine (A2R) and dopamine receptor heterocomplex [43]. Since the metabotropic glutamate receptors mGluR1/5 also contribute to this receptor interactome and their expression varies with the sleep–wake cycle, we further examined the complex formation between dopamine receptors and metabotropic glutamate receptors in both synaptic and extra-synaptic membranes. Furthermore, the metabotropic glutamate receptor mGlu1/5 clusters and directly interacts with the NMDA receptor in hippocampal neurons, reducing the NMDA receptor current and significantly decreasing the mGluR’s ability to release intracellular calcium through Homer protein and endoplasmic IP3R [44]. Therefore, immunoprecipitation experiments were conducted to clarify the roles of these proteins within the heterocomplex. Following sleep deprivation, protein levels related to immobilized anti-GluN2A and anti-GluN2B antibodies were evaluated using Western blotting. Since mGluRI, unlike mGluR5, is preferentially phosphorylated by the neurotrophic factor receptor, this modulation significantly influences mGluRI-dependent effects on neurotransmission, neuronal excitability, synaptic plasticity, and learning and memory processes [45]. Therefore, we analyze the protein interactome associated with mGluR1 rather than mGluR5.

**Figure 4:**
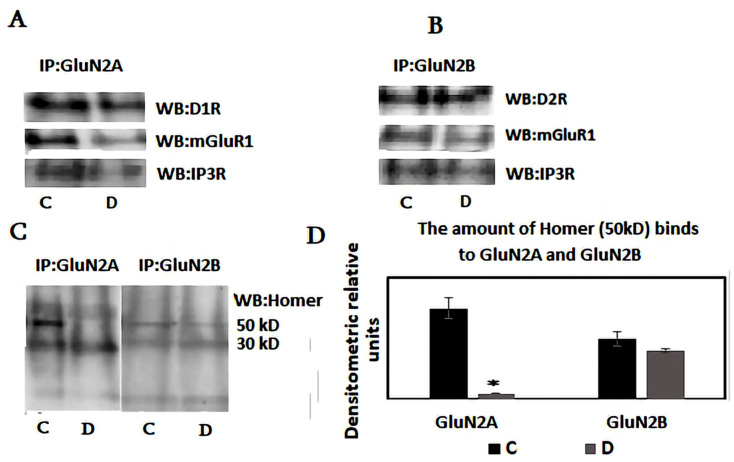
Western blot analysis was performed on proteins bound to anti-GluN2A and anti-GluN2B antibodies in the synaptic membranes of the hippocampus from control (n = 4) and sleep-deprived rats (n = 4). (**A**) Western blot analysis of proteins bound to the anti-GluN2A antibody; (**B**) Western blot analysis of proteins bound to the anti-GluN2B antibody; (**C**) Western blot analysis of Homer-positive proteins bound to anti-NR2a and anti-NR2b antibodies. Data are representative of three independent experiments. (**D**) Diagram displaying the densitometric analysis of Homer1a binding to the NR2a and NR2b antibodies in the synaptic fraction from hippocampal cells of control and sleep-deprived rats. Error bars represent one standard error of the mean, and asterisks indicate statistically significant differences: * *p* < 0.05; D (sleep deprivation) vs. C (control).

The immunoprecipitation experiments indicate that the levels of NR2A and NR2B in both synaptic and extra-synaptic fractions remain unchanged after SD (Figure 4A,B). The amounts of D1R and mGluR1 receptors associated with NR2A and NR2B also showed no alterations. However, sleep deprivation significantly reduced Homer and IP3R binding to NR2A in synaptic membranes, while no corresponding changes were observed in the NR2B-associated interactome (Figure 4C,D). These findings suggest that the protein supercomplex formed during wakefulness, comprising GluN2A, GluR1, IP3R, and Homer1, is disrupted during sleep deprivation. These alterations do not involve a quantitative redistribution of D1R, mGluR1, or GluN2A. The protein supercomplex involving the GluN2B subunit of the NMDA receptor and the D2 dopamine receptor is distinctly altered. In this heterocomplex, the dissociation of Homer and IP3R does not occur during sleep deprivation. Sleep deprivation increases the production of short Homer, which replaces long Homer on the GluN2A, mGluR1/5, and D1R hypercomplex, resulting in its dissociation. This data supports the suggestion about the role of splice variants of Homer1 and mGluR1/5 signaling in homeostatic sleep drive and output [18].

## 4. Discussion

Numerous studies indicate that sleep promotes memory consolidation, while sleep deprivation significantly impairs synaptic activity and cognitive functions through molecular mechanisms that are not yet fully understood [46,47]. Throughout the sleep–wake cycle, synapses undergo changes in composition and signaling, particularly in the strength of synaptic transmission, due to the redistribution of various postsynaptic receptors, including glutamate receptors [20]. AMPA and NMDA receptors, which are crucial ionotropic glutamate receptors in the central nervous system, facilitate fast excitatory neurotransmission and are essential for normal brain function [48]. Fluctuations in the expression of AMPAR during sleep and waking are related to learning and synaptic potentiation during wakefulness and synaptic weakening during sleep [49]. During sleep, the expression of the GluA1 subunit of AMPAR at synapses decreases, either because GluA1 subunits are removed from the surface or due to the substitution of GluA1 with weaker GluA2 subunits.

Despite some progress in this area, the precise molecular mechanism underlying the enhancement of synaptic activity during sleep deprivation remains unclear [50]. The internalization of AMPAR in postsynaptic areas and the mobility of AMPAR and NMDAR between synaptic and extra-synaptic sites may contribute to the observed synaptic strengthening [2]. Based on these observations, we examined the distribution of AMPA and NMDA receptors in the synaptic and extra-synaptic membranes following sleep deprivation. Our findings indicate that six hours of sleep deprivation increases the synaptic content of the AMPAR subunit GluA1, while the amount of this subunit decreases in the extra-synaptic fractions. These results align with recent observations that prolonged wakefulness and sleep deprivation (SD) are associated with enhanced glutamatergic synaptic strength [51,52] and a greater number of functional AMPA receptors (AMPARs) in synapses [53,54]. Since the recruitment of AMPA receptors and the increase in their numbers at the synapse are primary mechanisms mediating synaptic plasticity [16], these data suggest that sleep deprivation may enhance brain homeostasis. Our results also indicate that, unlike the AMPA receptor, the levels of NMDAR subunits do not significantly change their location after six hours of sleep deprivation. The GluN1, GluN2A, and GluN2B amounts did not change substantially in synaptic or extra-synaptic membranes after SD. The limited mobility of NMDAR may reduce its ability to trigger NMDAR-dependent long-term potentiation at these synapses, resulting in memory deficits associated with sleep deprivation. This suggests that the alterations in synaptic plasticity caused by sleep deprivation are likely due not to a quantitative shift of NMDAR glutamate receptors but to changes in the activity of synaptic proteins and post-translational modifications related to glutamatergic signaling transmission.

The trafficking of glutamate receptors relies on the dynamics of the actin cytoskeleton, which enhances the expression of AMPA receptors at the postsynaptic membrane [37,38,39]. We examined actin dynamics during sleep deprivation by analyzing globular (G) and filamentous (F) actin in brain samples. Our findings revealed a decreased G-to-F ratio, indicating that the actin cytoskeleton undergoes polymerization during sleep deprivation. Additionally, the assembly of actin filaments correlated with cofilin activation, which is dephosphorylated during sleep deprivation. Furthermore, we evaluated the actin-regulating protein Rho. We found that Rho showed a connection with the scaffold protein interactome, with its levels decreasing during sleep deprivation. Rho GTPases regulate LIM-kinase activity, which phosphorylates cofilin, transforming it into an inactive form [55]. Therefore, sleep deprivation leads to the assembly of the actin cytoskeleton, enhancing the dynamics and mobility of postsynaptic components while increasing hippocampal spine density. Interestingly, the organization of the actin cytoskeleton occurs when the AMPA receptor subunit is displaced from the extra-synaptic to the synaptic region. Consequently, actin complex polymerization coincides with the incorporation of AMPA receptors into the synaptic membrane.

In addition to glutamatergic signaling, dopaminergic transmission is crucial for protein synthesis-dependent types of synaptic functions in the hippocampus. However, the molecular mechanisms by which dopamine influences synaptic plasticity remain poorly understood. The locus coeruleus neurons may be involved in the dopaminergic modulation of memory consolidation. Dopamine-releasing neurons originate in the locus coeruleus and project to the hippocampus, facilitating and enhancing memory consolidation. Consequently, dopaminergic neurons may bolster memory after encoding, which aligns with the potential co-release of dopamine in the hippocampus [14]. The activation of dopamine receptors increases the GluA1 subunit of AMPA receptors, resulting in a greater incorporation of surface GluA1 at synaptic sites [56,57]. Since inserting GluR1 into synaptic membranes is associated with an increased frequency of miniature synaptic events, these findings suggest that local dopamine-dependent protein synthesis could modify and activate ‘silent’ synapses. Given that ‘silent’ and active synapses can fluctuate during the sleep–wake cycle, it is essential to investigate the role of dopamine receptors in regulating sleep phases and the transition between sleep and wakefulness.

Protein–protein interactions and changes in the protein interactome formation in the postsynaptic membranes could play crucial roles in modulating glutamatergic signal transduction during sleep deprivation. Therefore, the interaction of proteins and the resulting protein interactome within the postsynaptic membranes may be significant for glutamatergic signaling [58]. Changes in protein–protein interactions within the synaptic membranes during sleep deprivation and transitional sleep phases are poorly understood. Both dopamine receptors, D1 and D2, can bind to several synaptic proteins, including glutamate receptors. D2 receptors directly interact with GluN2B, which is essential for modulating NMDAR-mediated currents and behavioral responses to dopamine [59]. The D1 receptor interacts with the GluN2A subunit of the NMDAR through amino acid sequences located in their respective intracellular C-tails, and this interaction regulates the surface dynamics and distribution of both receptors [60,61,62]. Uncoupling the D1 and NMDA receptor complex alters NMDA-dependent long-term potentiation, resulting in working memory deficits [63]. This weak and transient association between NMDAR and D1 receptors can significantly influence the structural and functional roles of synaptogenesis within the hippocampus [64]. The GluN2A subunit of NMDAR can also form a heterocomplex with several scaffold proteins, such as the PSD-MAGUK family, and various G protein-coupled receptors (GPCRs), including adenosine receptors and mGluR1/5 [45,65,66,67]. In this study, we analyzed the protein interactome involving the GluN2A and GluN2B subunits of the NMDA receptor. The findings indicate that sleep deprivation reduced the binding of Homer and IP3R to the GluN2A/D1R/mGluR heterocomplex, while the levels of D1R and mGluR1 in the protein interactome remained unchanged. In contrast, the change in Homer and IP3R content does not occur in the GluN2B/D2R/mGluR1 complex, which may indicate that only the heterocomplex involving NMDA and D1 receptors is sensitive to sleep deprivation.

Thus, these data suggest that sleep deprivation dissociates the heterocomplex connecting plasma membrane glutamate receptors with the endoplasmic sites via long Homer1. This disorganization is likely caused by the increased synthesis of short Homer1a during sleep deprivation, which replaces the long Homer only in the GluN2A/D1R/mGluR1/IP3R heterocomplex. Disruption of this protein interactome is expected to diminish glutamate-dependent calcium release from the endoplasmic reticulum and decrease Ca-dependent protein synthesis. These data support the suggestion of an activity-dependent dissociation of the Homer1-containing interactome by substituting a short splice variant of Homer [68]. However, it remains unclear how D1 receptor activity changes following the dissociation of this complex. Synergistic receptor–receptor interactions occur in the D1-NMDA heterodimer, where NMDA receptor activation can recruit D1 receptors to the plasma membrane, enhancing D1 signaling and cAMP accumulation [60]. This reciprocally facilitating positive feedback loop, if left unregulated, has the potential to cause simultaneous overactivation of both D1 and NMDA receptors, which may lead to neurotoxicity. PSD-95 inhibits D1–NMDA receptor association and uncouples the NMDA receptor-dependent enhancement of D1 signaling [69]. Thus, protein trafficking and membrane clustering are essential for regulating NMDAR-D1R interactions. Structural and post-translational modifications, such as palmitoylation of the N-terminal regions, significantly regulate PSD-95, alter protein localization, and specifically modify glutamate receptor function and neuronal excitation [33]. Our further research focuses on clarifying the role of PSD-95 in the alterations of protein–protein interactions involving dopamine and glutamate receptors.

## 5. Conclusions

The association between glutamate and dopamine receptors, as well as the Homer1 scaffold protein, in synaptic and microsomal membranes was evaluated during 6 h periods of sleep deprivation. Our findings revealed that sleep deprivation causes the dissociation of the Homer complex from the IP3R and mGluR5 in microsomal membranes. Furthermore, the analysis of the synaptic membrane protein interactome indicates that sleep deprivation decreases the binding of Homer and IP3R to the GluN2A/D1R/mGluR1 heterocomplex, whereas the levels of GluN2A, D1R, and mGluR1 remain unchanged. In contrast, the alteration in Homer and IP3R content does not occur within the GluN2B/D2R/mGluR1 complex, suggesting that only the heterocomplex involving the GluN2A subunit of NMDA and D1 receptors is sensitive to sleep deprivation. Consequently, sleep deprivation leads to the redistribution of Homer proteins, affecting the synaptic and endoplasmic membrane protein interactome involving GluN2A, mGluR1/5, and D1 receptors. This evidence supports the idea that Homer1 has a regulatory role in glutamate and dopamine receptor signaling during sleep–wake cycles.

## Data Availability

The original contributions presented in this study are included in the article/Supplementary Materials. Further inquiries can be directed to the corresponding author(s).

## Supplementary Materials

The following supporting information can be downloaded at: https://www.mdpi.com/article/10.3390/neurosci6030061/s1, File S1: Original Western Blot Figures (neurosci-06-00061).

## Abbreviations

SD	Sleep deprivation
NMDA	N-Methyl-D-Aspartate
AMPA	α-Amino-3-hydroxy-5-methyl-4-isoxazolepropionic acid
mGluR1/5	Metabotropic glutamate receptor type 1
IP3R	Inositol trisphosphate receptor
DA	Dopamine
D1R	Dopamine1 receptor
D2R	Dopamine2 receptor
PSD	Postsynaptic density
GRIP	Glutamate receptor-interacting protein
LTD	Long-term depression
LTP	Long-term potentiation
GluN2A	GluN2A subunit of NMDA glutamate receptor
GluN2B	GluN2B subunit of NMDA glutamate receptor
GluN1(NR1)	GluN1 subunit of NMDA glutamate receptor
