# Supplementary material for: Sleep Deprivation in Rats Causes Dissociation of the Synaptic NMDA Receptor/D1 Dopamine Receptor Heterocomplex
Source: NeuroSci. 2025 Jul 5;6(3):61. doi: 10.3390/neurosci6030061 (PMC12286143; doi:10.3390/neurosci6030061)
Supplement: Supplementary file 1 [file neurosci-06-00061-s001.zip › neurosci-3720037-supplementary.pdf]

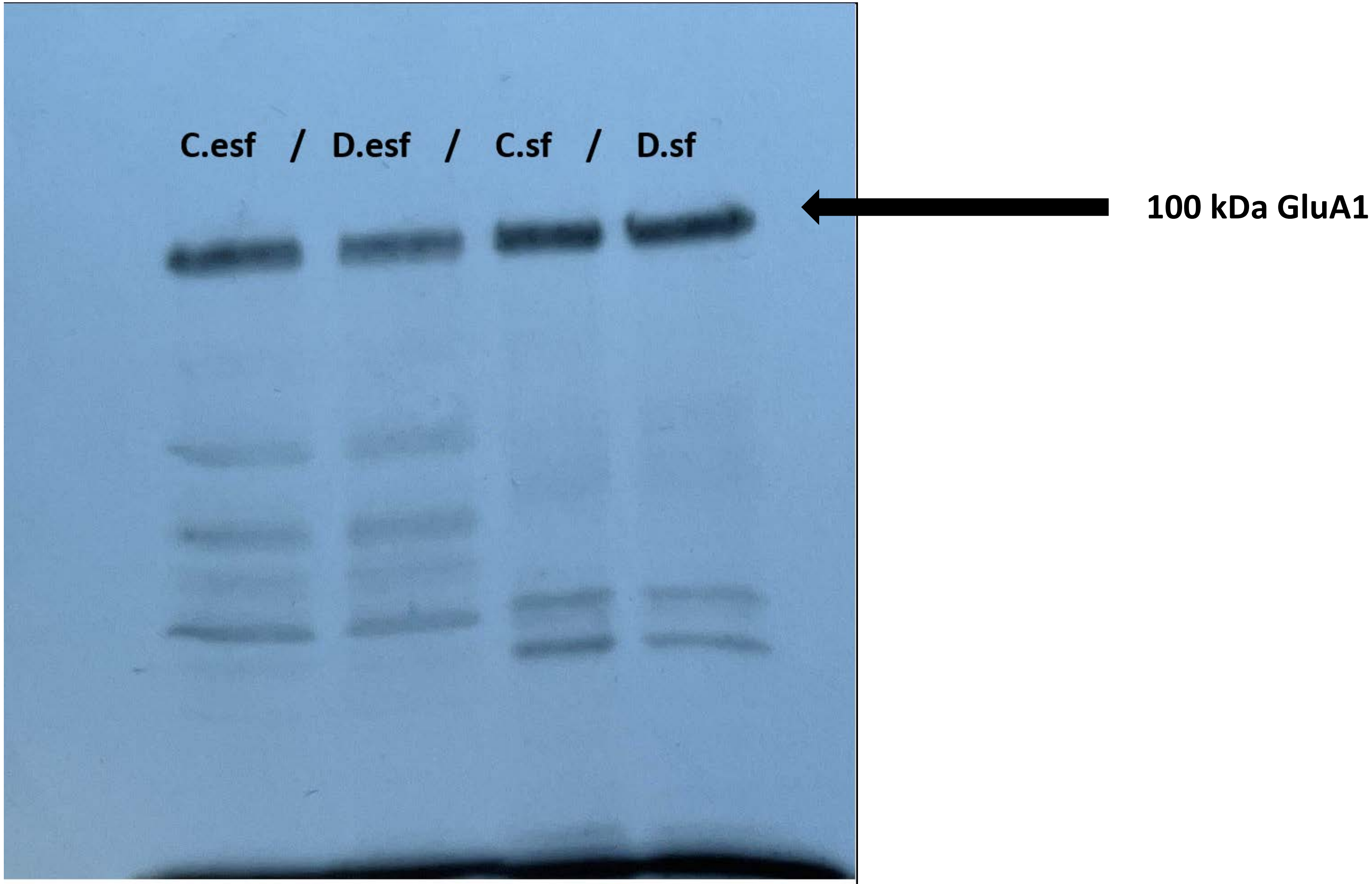

Figure 1(A) – GluA1

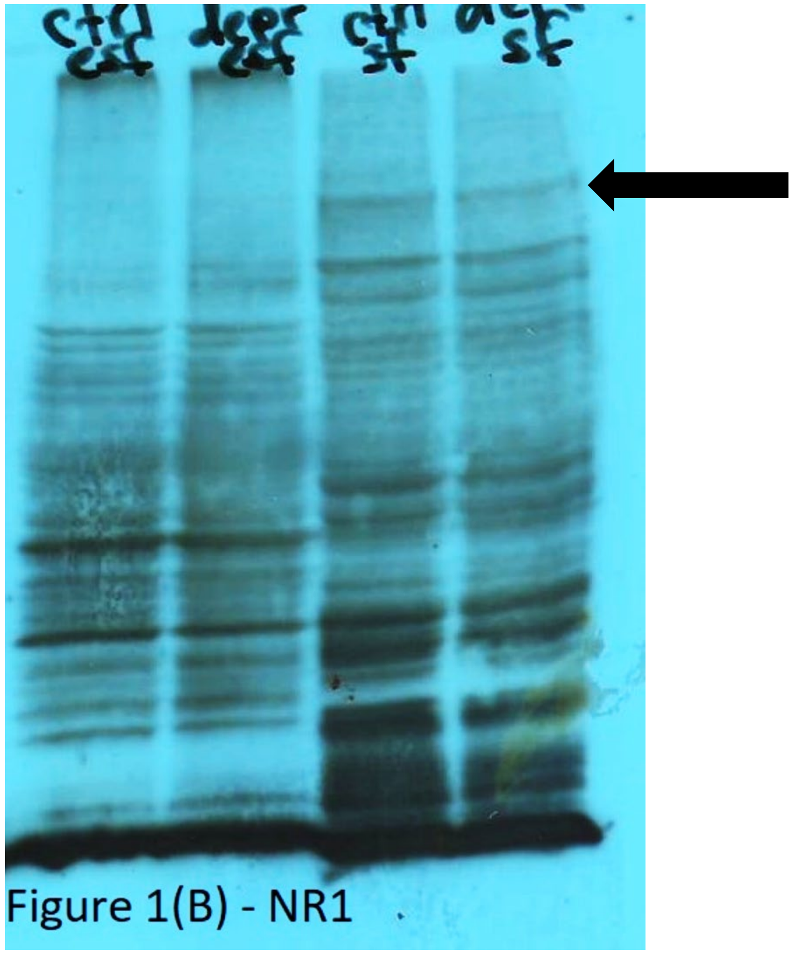

Figure 1(B) - NR1

Figure 1 (C)

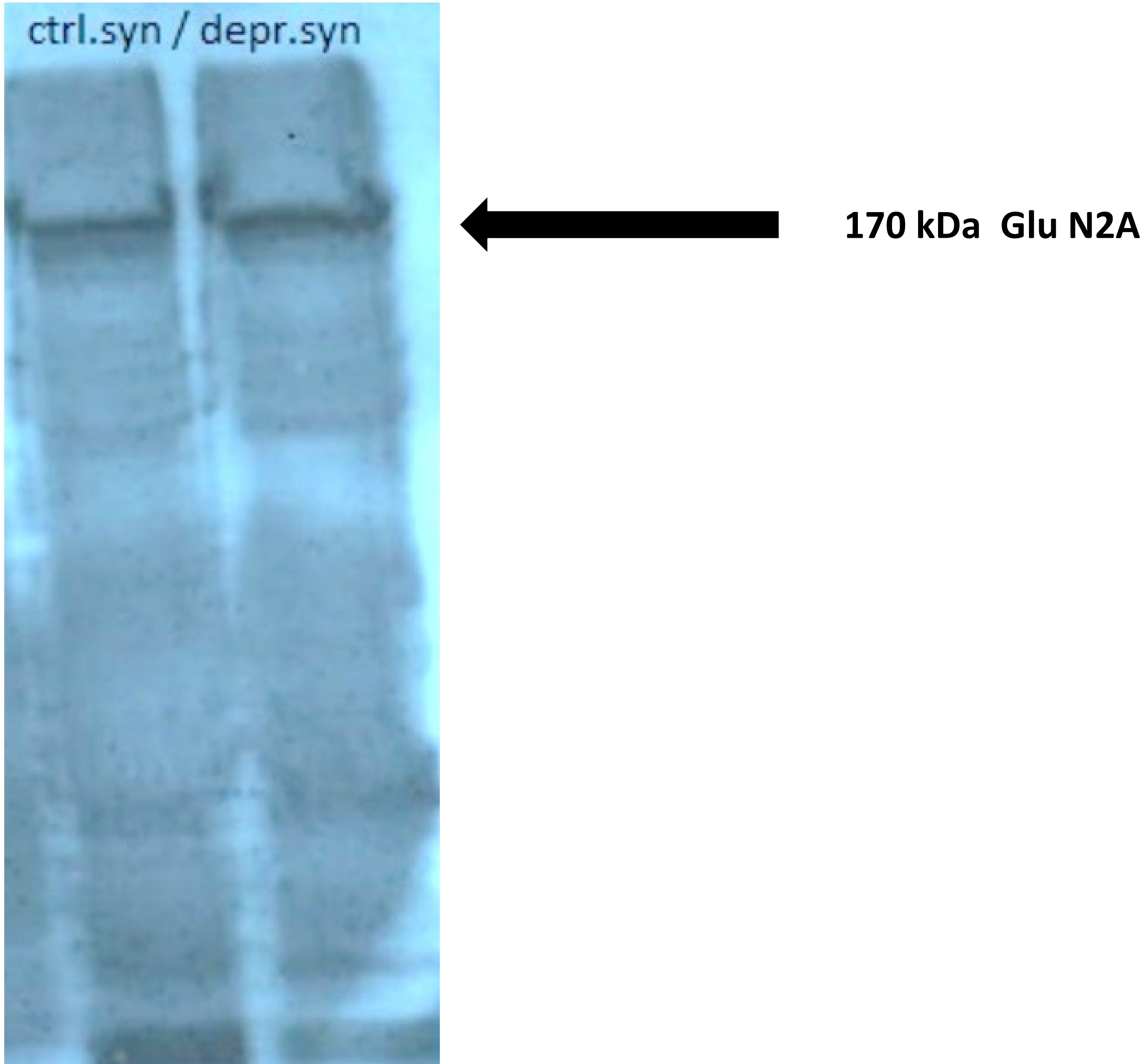

Figure 1 (C)

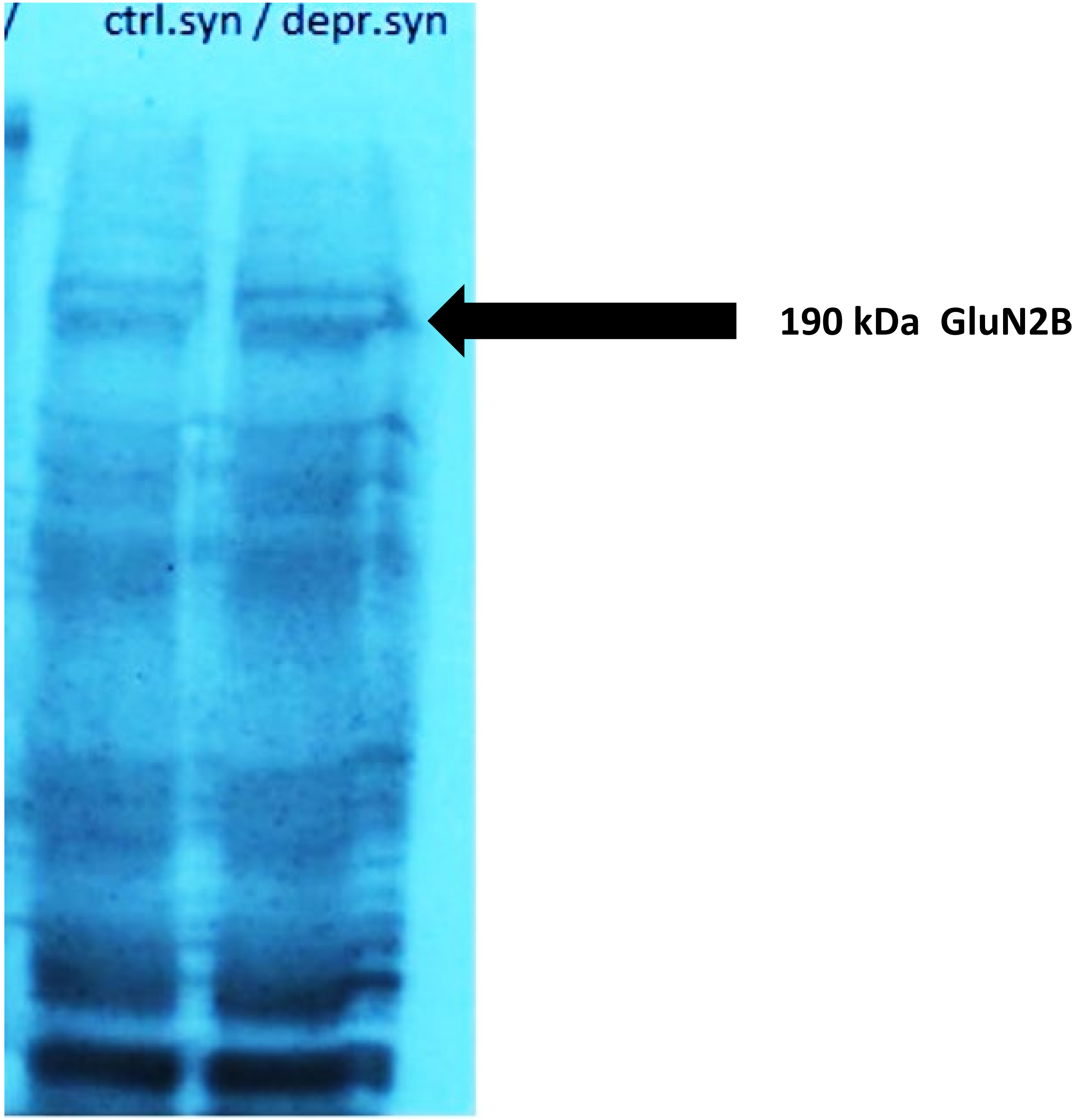

Figure 2 (A) G-F actin bands

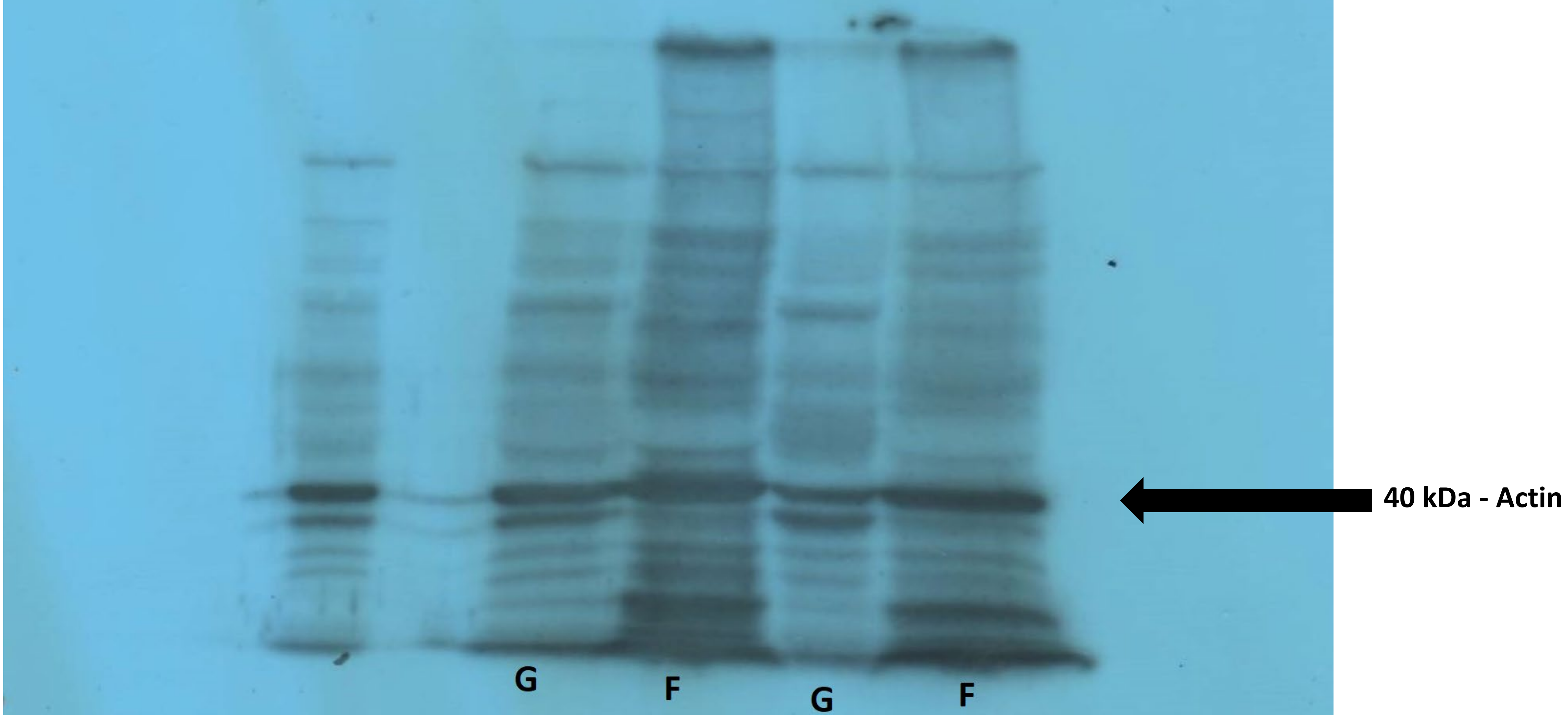

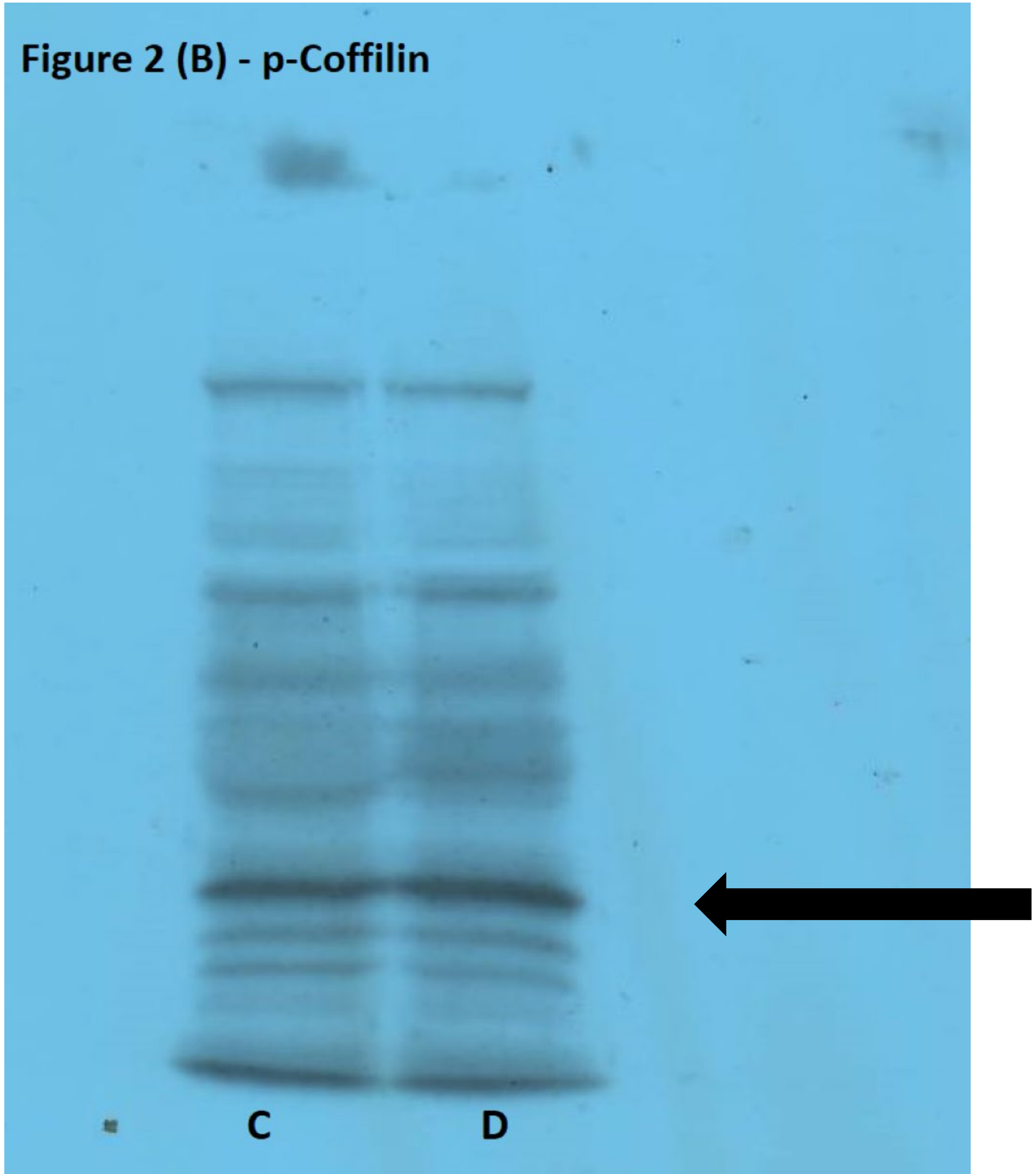

20 kDa – p-Coffilin

Figure 3 (A)

IP: Homer

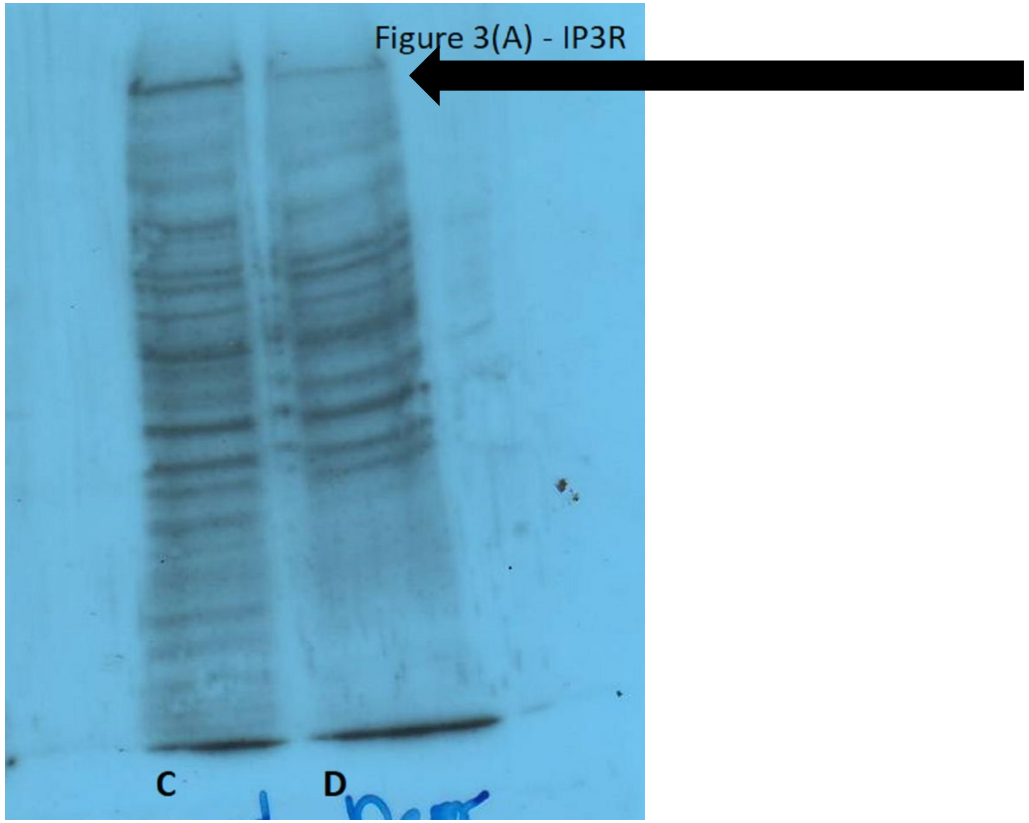

240 kDa- IP3R

Figure 3 (B)

IP: Homer

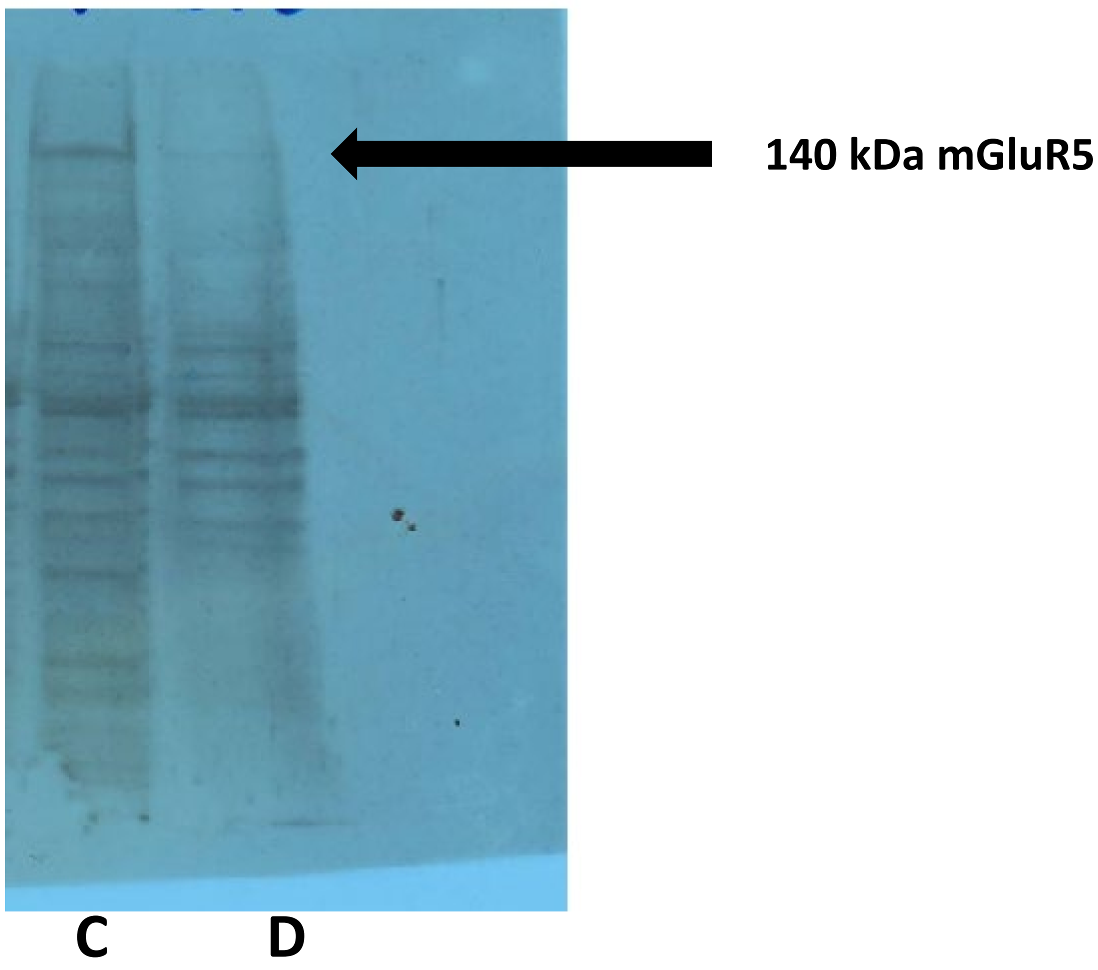

IP: GluN2A

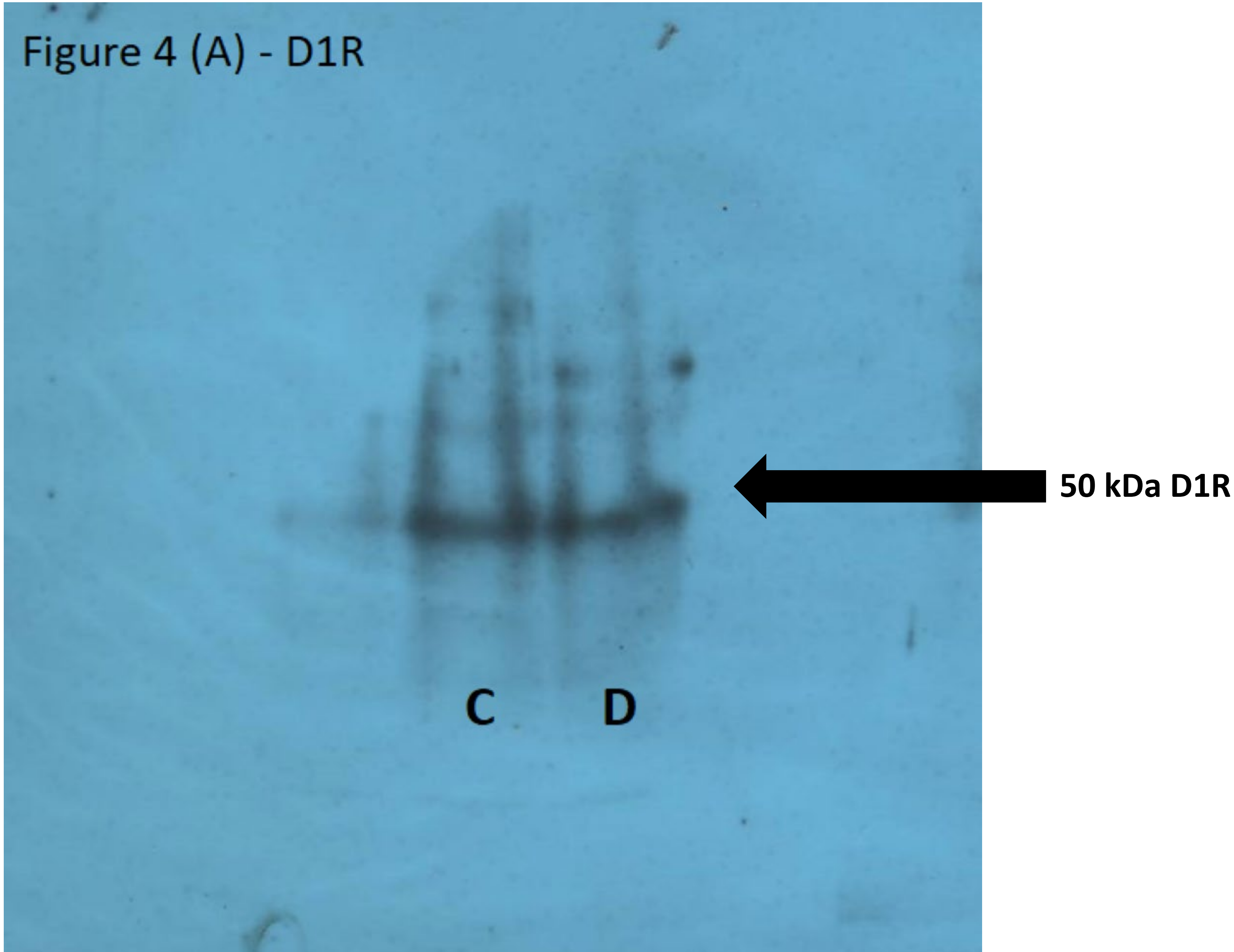

IP: GluN2A

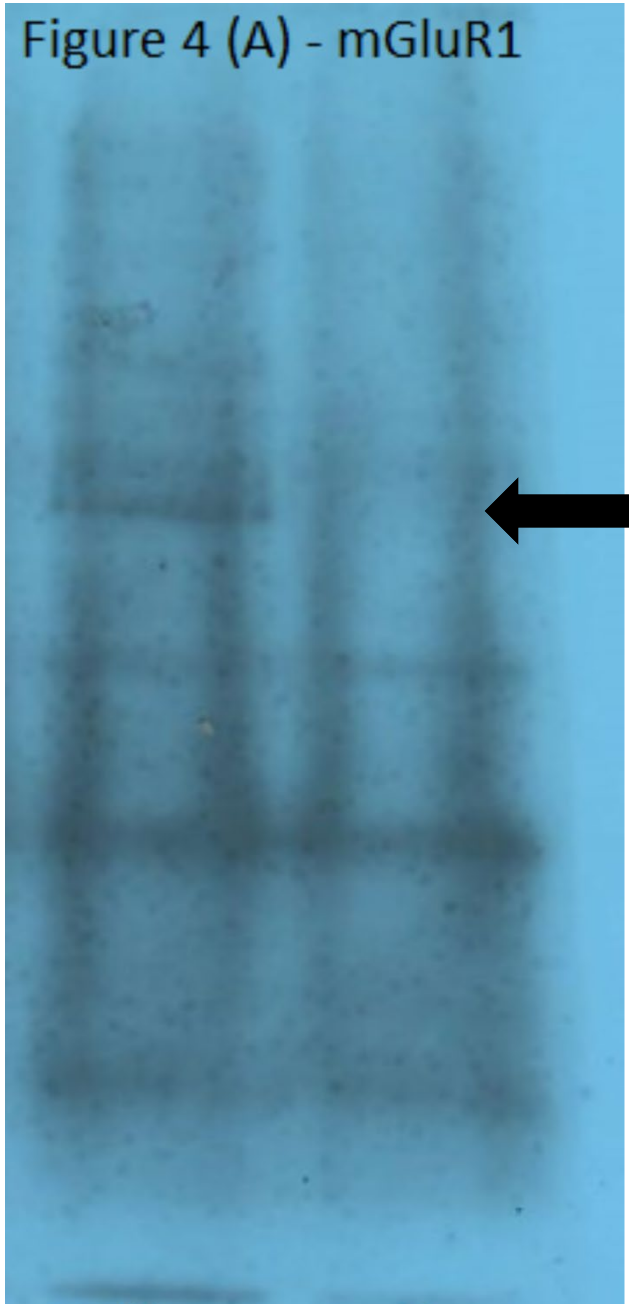

140 kDa – mGluR1

IP: GluN2A

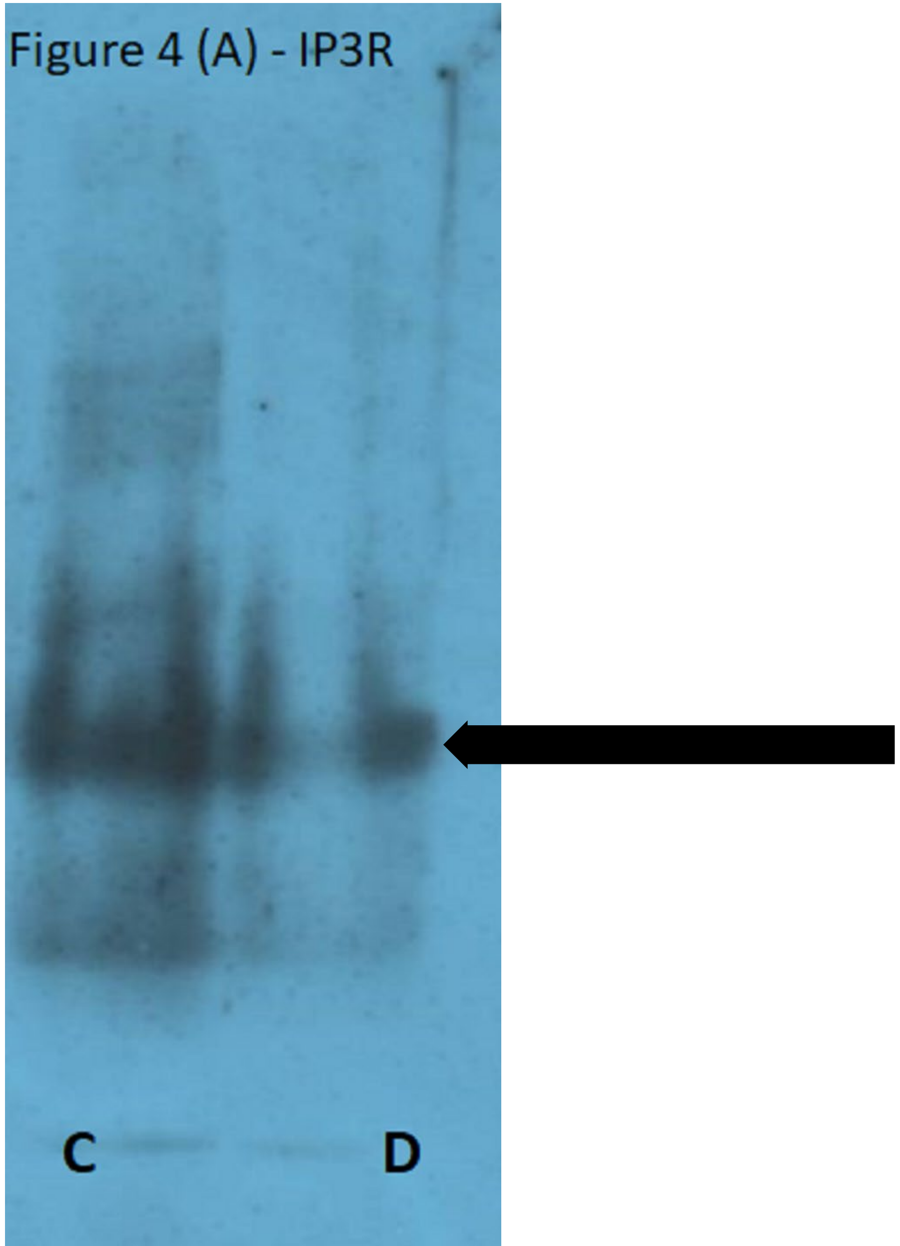

IP: GluN2B

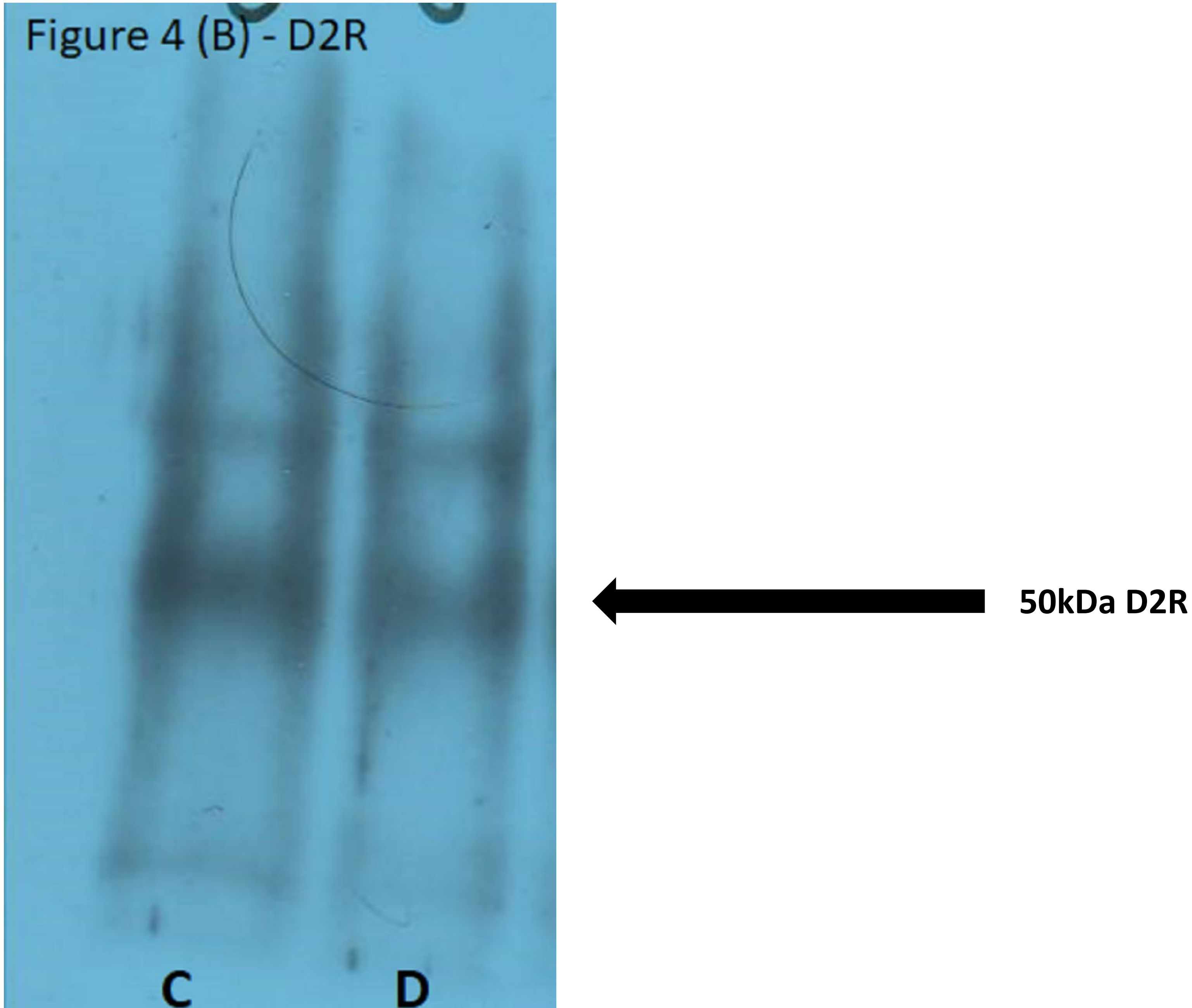

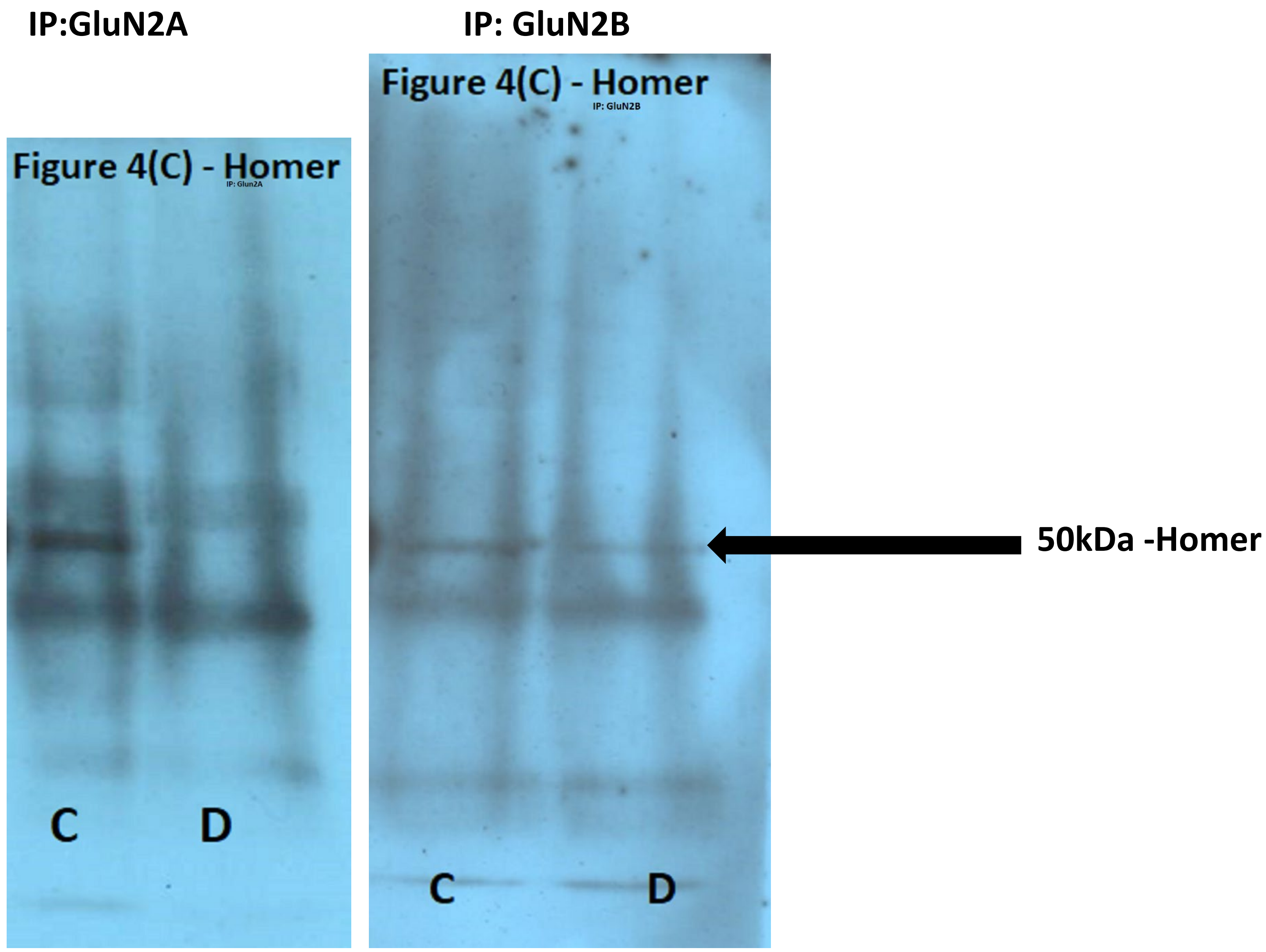
IP:GluN2A
IP: GluN2B
Figure 4(C) - Homer
IP: Glun2A
C
D
Figure 4(C) - Homer
IP: GluN2B
C
D
50kDa -Homer

Figure 3 (A)

IP: Homer

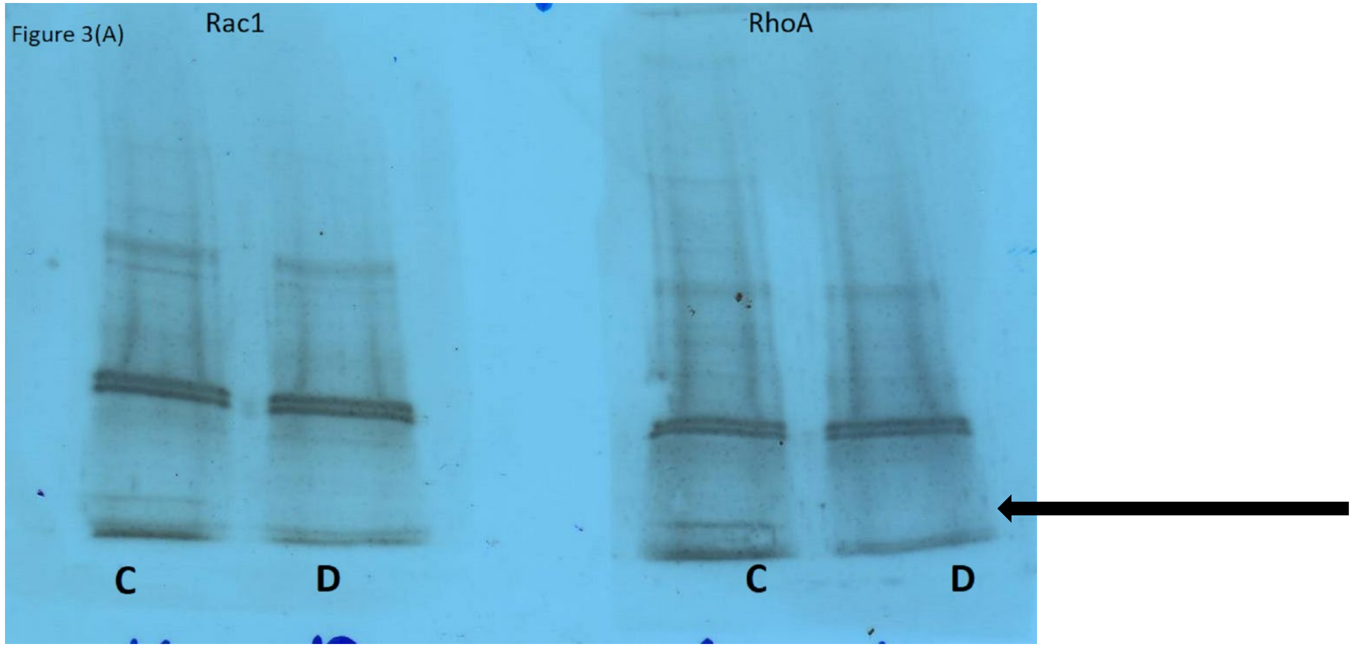

20 kDa -RhoA
